# What Do We Know about *Candida auris*? State of the Art, Knowledge Gaps, and Future Directions

**DOI:** 10.3390/microorganisms9102177

**Published:** 2021-10-19

**Authors:** Victor Garcia-Bustos, Marta D. Cabanero-Navalon, Amparo Ruiz-Saurí, Alba C. Ruiz-Gaitán, Miguel Salavert, María Á. Tormo, Javier Pemán

**Affiliations:** 1 Department of Internal Medicine and Infectious Diseases, University and Polytechnic La Fe Hospital, 56026 Valencia, Spain; salavert_mig@gva.es; 2 Severe Infection Research Group, Health Research Institute La Fe, 46026 Valencia, Spain; albacruiz@gmail.com (A.C.R.-G.); tormo_man@iislafe.es (M.Á.T.); peman_jav@gva.es (J.P.); 3 Department of Pathology, Faculty of Medicine and Dentistry, University of Valencia, 46010 Valencia, Spain; amparo.ruiz-sauri@uv.es; 4 Department of Medical Microbiology, University and Polytechnic La Fe Hospital, 46026 Valencia, Spain

**Keywords:** *Candida auris*, candidaemia, virulence, pathogenesis, epidemiology, diagnosis, antifungal agents, outbreak

## Abstract

*Candida auris* has unprecedently emerged as a multidrug resistant fungal pathogen, considered a serious global threat due to its potential to cause nosocomial outbreaks and deep-seated infections with staggering transmissibility and mortality, that has put health authorities and institutions worldwide in check for more than a decade now. Due to its unique features not observed in other yeasts, it has been categorised as an urgent threat by the Centers for Disease Control and Prevention and other international agencies. Moreover, epidemiological alerts have been released in view of the increase of healthcare-associated *C. auris* outbreaks in the context of the COVID-19 pandemic. This review summarises the current evidence on *C. auris* since its first description, from virulence to treatment and outbreak control, and highlights the knowledge gaps and future directions for research efforts.

## 1. Introduction

The genus *Candida* is composed of approximately of 200 species. It is the most important fungal genus in the medical field [1], as some *Candida* species are the main cause of worldwide invasive fungal infections (IFI) [2,3]. *Candida albicans*, *Candida glabrata*, *Candida parapsilosis*, *Candida dubliniensis*, *Candida tropicalis*, and *Candida krusei* are the most frequently isolated species in patients with IFI. Nevertheless, since its first description in 2009, a new *Candida* species that has simultaneously emerged in the five populated continents as a new and serious public health threat has ended up accounting for most of the *Candida* isolates [4,5] and IFI in some regions [6,7,8,9]: *Candida auris*.

*C. auris* is an emergent species which, as a consequence of its multidrug resistance to common antifungals [10,11,12], difficult identification with conventional biochemical microbiological techniques [13,14], high transmissibility, surface survival [15], and environmental adaptability [16,17], has been associated with serious nosocomial IFI with high mortality and is extremely difficult control in many countries [11,16,18,19,20].

All of these characteristics make it conspicuously different from the rest of the *Candida* species, and it constitutes the only fungal species able to be intrinsically resistant to the three main antifungals used in medical practice, namely azoles, amphotericin B, and even the first-choice agents for *Candida* invasive infections: echinocandins. In fact, in 2019, the Centers for Disease Control and Prevention of the United States (CDC) considered *C. auris* infection an urgent threat for international public health in the field of multidrug resistant microorganisms [21,22]. Moreover, in our actual context of the SARS-CoV-2 pandemic, its incidence seems to be increasing due to outbreaks in specialised COVID-19 treatment units [23,24,25,26,27,28,29,30,31].

## 2. Importance and Chronology of *C. auris* Emergence

*C. auris* was first isolated in the ear of a Japanese patient with external otitis in 2009 [6]. Since then, hospital outbreaks and IFI caused by this species have been described in more than 40 countries in the five populated continents, creating a global health problem. Due to its high multidrug resistance, transmissibility, ability to indefinitely colonise patients, and long persistence in the hospital environments, it has alerted the health authorities and health organisations of America and Europe. 

In June 2016, the CDC communicated an extraordinary clinical alert, warning U.S. health institutions of the global emergence of *C. auris* and its capacity to cause serious IFI outbreaks in U.S. health centres [32]. Only one week after this CDC warning, Public Health England announced the isolation of this pathogenic fungus in hospitals in the United Kingdom, and reported a non-controlled outbreak of nosocomial candidaemia in the Royal Brompton Hospital in London [33], which preceded the notification in Spain of the largest European outbreak in Valencia, in the University and Polytechnic Hospital La Fe. 

In October of that same year, the Pan American Health Organization (PAHO/WHO) also issued warnings about *C. auris*, and issued a new epidemiological alert about the risk of new nosocomial outbreaks in Latin America, recommending that Member States build capacity for early detection and effective reporting to prevent and control its spread in health services [34]. At the end of December 2016, when the nosocomial outbreaks in London and Valencia affected almost 100 patients, the European Centre for Disease Prevention and Control (ECDC) warned of the emergence of *C. auris* in Europe, and published a *Rapid Risk Assessment* update, appraising the risk for its spread in hospitals in European Union and European Economic Area (EU/EEA) countries [32]. 

Since then, the frequency of notifications of IFI due to *C. auris* has been increasing worldwide. In 2019, in the *Report on Urgent Threats* from the CDC, *C. auris* was again categorised as one of the main urgent threats, together with carbapenem-resistant *Acinetobacter baumanii* and Enterobacteriaceae, *Clostridioides difficile*, and *Neisseria gonorrhoeae*, with priority over other well-known resistant pathogens such as Enterobacteriaceae with extended-spectrum beta-lactamase (ESBL) production, methicillin-resistant *Staphylococcus aureus* (MRSA), and multidrug resistant *Pseudomonas aeruginosa* [21].

Recently, the use of personal protective equipment (PPE) in the SARS-CoV-2 pandemic has not helped to control *C. auris* transmission. In fact, many *C. auris* outbreaks have been described in COVID-19 units, both in critically ill units and conventional hospital wards. Until now, outbreaks have been identified in the USA [23,24], Italy [25], Colombia [26], India [27], Mexico [28], Lebanon [29], Brazil [30], and Spain [31].

Due to its nosocomial transmission and its ability to easily colonise the hospital environment, the SARS-CoV-2 pandemic has created an ideal atmosphere for *C. auris* dissemination. The hospital saturation, the equipment used, and the decreased efficacy of microbiology prevention systems are some of the main reasons for the increased *C. auris* spread during the actual SARS-CoV-2 pandemic, especially in developing countries [35].

## 3. Hypotheses on the Origin of *C. auris*

Since its first isolation in Japan almost a decade ago [6], one of the most enigmatic traits of *C. auris* has been the almost simultaneous and independent emergence of isolates of different clonality, as demonstrated by whole genome sequencing (WGS) studies [19]. Despite *C. auris* being detected retrospectively in several cases both from colonization and invasive samples, mainly in South Korea, the absence of this yeast in collections going back several decades was not due to identification problems [36]. After the first reports of cases of invasive infection in patients from Asia, Africa, and South America with strains belonging to phylogenetically different clades [19,37,38,39,40], *C. auris* began to be considered a pathogen of medical importance in humans. However, the mechanisms underlying the appearance of highly virulent and resistant strains in geographically distant regions without phylogenetic traceability since the first descriptions in the literature are still unknown.

The indiscriminate use of antifungal agents both in clinical practice and agro-industry has been proposed to contribute to the emergence of *C. auris*, and may partially explain its high degree of drug resistance [11]. Nevertheless, this hypothesis hardly justifies its appearance as a virulent human pathogen on three continents almost simultaneously [41], nor does its significant pathogenicity both in humans and in other animal experimental models [11,16,18,19,20,42,43].

Another suggested explanation for the emergence of *C. auris* and for its unusual characteristics has been the recent and progressive acquisition of virulence factors [42]. But, similarly, it is unlikely that these determinants of pathogenicity have been acquired nearly simultaneously in separated remote regions under different environmental and genetically distant isolates [41,44].

Recently, global warming has been postulated as a feasible explanation for this unknown [41,44,45]. Of the large number of fungal species described in our planet, only a minority are human pathogens, mainly due to the high basal body temperature of mammals, which created a thermal restriction barrier, as well as the complex mechanisms of innate and adaptive immunity against fungal infection [46,47].

Casadevall et al. compared thermal sensitivity of *C. auris* with other closely phylogenetically related *Candida* species, and demonstrated its relatively high thermotolerance [41]. Hence, it was hypothesised that *C. auris* could have overcome the thermal barrier of mammals, as a result of its adaptation to global warming and higher temperatures from an environmental reservoir, possibly in wetlands or coastal ecosystems. Later, it could have been transported by migratory animals such as birds to other areas of the planet where, after interspecific transmission in rural areas, human colonization and its subsequence appearance in healthcare facilities could take place. The recent environmental isolation of *C. auris* in tropical remote beaches of the Andaman Islands (India) [48] confirms for the first time the presence of an environmental niche and supports the global warming hypothesis in the emergence of *C. auris*.

## 4. Microbiological Features of *C. auris*

### 4.1. Phylogeny 

*C. auris* is an ascomycete fungus within the clade *Clavispora* of the family *Metschnikowiaceae* and *Saccharomycetales* Order [49,50]. Although the evolutionary phylogenetic relationship of *C. auris* with other *Candida* species is not yet fully clarified due to the infrequency of some of the closest species, 5 clades have been described so far. These clades have been related to other species such as such as *C. haemulonii*, closely followed by *C. pseudohaemulonii*, and *C. dobushaemulonii* with 88% similarity [49,51], and recently, *C. heveicola* [6]. 

Due to the relative taxonomic proximity of these species, *C. auris* shares some of their phenotypic characteristics, preventing an adequate identification based on conventional biochemical methods [52].

Whilst clades I, III and IV are responsible for outbreaks of invasive infection by multidrug resistant strains, the clade II located geographically in east Asia has not been associated with nosocomial outbreaks. It presents a more benign antifungal drug susceptibility profile, a markedly different karyotype from the rest, and has been fundamentally described in ear infections, as it was at the time of its discovery [6,53,54]. Clade V, recently described in Iran [39], is highly infrequent, and owns a high degree of phylogenetic proximity with clades I, III and IV, although its sequence is relatively divergent from the rest [53]. Each of these clades presents isolates of the same clonality, restricted to a specific geographical area, but which historically emerged in a relatively simultaneous and independent manner [17], as previously discussed. 

Clade I has been described mainly in regions of the United Kingdom, India, and Pakistan. Clade II is found mostly in Japan and South Korea. Clade III is native to South Africa, and also includes samples from Spanish outbreaks, while clade IV constitutes that described in Venezuela. Finally, clade V has been described in Iran, with a single isolate from a patient who never left the country [6,17,19,20,49,53,54,55].

### 4.2. Culture, Growth, and Phenotypes

*C. auris* is able to grow after 24 h of culture at 37 °C on Sabouraud agar, where it develops opaque white to creamy colonies. Chromogenic media have recently become popular for *C. auris* culture and identification. In the medium CHROMagar Candida^®^, colonies present with pink to pale purple tonalities. However, differences in the tone of the colonies have been reported, dependent on the country of origin and clade. Some authors have, hence, proposed these chromogenic media complemented with Pal agar (with extract of sunflower seeds) for presumptive identification of *C. auris* [56]. 

Although it is not able to grow in media with cycloheximide, *C. auris* presents a marked thermotolerance and salt tolerance, growing in a temperature range from 37–42 °C, unlike other *Candida* or fungal species [15,20,41,43,49,57,58,59,60]. These particular traits, beyond modified chromogenic media, can also be used for its presumptive identification in microbiology laboratories with technical limitations or before definite molecular identification. 

*C. auris* assimilates and weakly ferments glucose, saccharose, and trehalose; and assimilates raffinose, melezitose, soluble starch, and ribitol or adonitol. However, it is not capable of fermenting galactose, maltose, lactose, or raffinose [6]. This glycidic fermentation and assimilation profile also makes it possible to generate sensitive and specific culture media based on mannitol, dextrose, and dulcitol to isolate and presumptively identify *C. auris* in clinical practice [15].

Microscopically, *C. auris* is a yeast with 2–3 × 2.5–5 μm ovoid cells similar to *C. glabrata* [43]. It presents two important clearly distinguishable phenotypes with different behaviour and virulence [43,61,62,63,64,65,66,67,68,69]:

- Non-aggregative phenotype: yeast cells arrange as isolated or, sometimes, coupled cells, similarly to other *Candida* species.
- Aggregative phenotype: some isolates keep daughter cells attached after budding, creating large aggregates that cannot be separated by physical disruption after vigorous vortexing for several minutes.

The different characteristics in behaviour, virulence, and pathogenicity determinants of both phenotypes will be posteriorly discussed.

Unlike other species of the genus *Candida*, such as *C. albicans*, considered the most virulent species of the group, and with high filamentation capacity [70,71,72], *C. auris* is not considered able to develop true hyphae, chlamydospores, or germ tubes [34,36,58,73,74]. The formation of very rudimentary pseudohyphae had only been described occasionally [43,62]. However, more recent studies have reported filamentation in some strains of *C. auris* under certain environmental conditions or stress [61,71,75,76]. Yue et al. described an in vivo inheritable phenotypic change or switch towards a filamentous or filamentation-competent phenotype, induced by passage through the mammalian organism, different salt concentrations of NaCl between 10% and 26%, and thermal changes [75]. Our group recently described filamentation in non-aggregative and aggregative strains in an invertebrate model in wax moth larvae at 37 °C [61]. On the other hand, Bravo-Ruiz et al. were able to induce filamentation in vitro through genotoxic stimulation [76]. This possibility of pseudohyphae formation has finally been demonstrated in strains from the four main clades, according to the work of Fan et al. [77].

### 4.3. Difficulties in C. auris Identification

**Figure 1:**
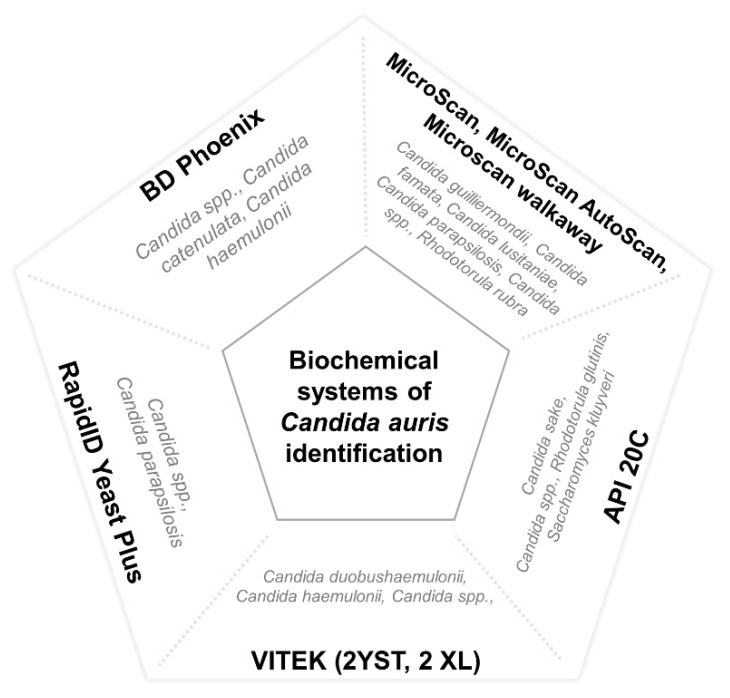
Main misidentified species of different commercial biochemical systems. Misidentification of *C. auris* by means of the VITEK systems has specially been reported with isolates of the east Asian and African clades.

There are numerous methods used for the identification of *Candida* species in clinical microbiology laboratories. Nevertheless, most of them use commercial systems of biochemical characterization, which are unable to properly identify *C. auris*. These methods usually misidentify it as *C. haemulonii*, *Rhodotorula glutinis*, *Saccharomyces cerevisiae*, or, less frequently, as other Candida species such as *C. famata*, *C. dobushaemulonii*, *C. sake*, *C. lusitaniae*, *C. albicans*, *C. guilliermondii*, or *C. parapsilosis* [17,18,43,52,57,58,73,78,79,80,81,82,83]. However, erroneous identification has been reported with more complex diagnostic methods, such as filmarray systems [84] and matrix-assisted laser desorption/ionisation time-of-flight (MALDI-TOF) [85]. The main misidentified species of different commercial biochemical systems is represented in Figure 1.

In primary or secondary hospitals with fewer resources, as well as in developing countries with limited access to sophisticated and expensive methods such as MALDI-TOF or molecular techniques, such identification sometimes arrives at *Candida* spp. without reaching the species level in non-invasive samples [19,86]. However, due to its relevance for public health, accurate and rapid diagnostic methods are needed to facilitate prompt diagnosis, effective patient management, and control of nosocomial outbreaks.

At present, the new MALDI-TOF systems, after including the specific spectra in the databases [87,88,89], are able to provide specific diagnoses at species level. In developing countries with limited access, this method could be replaced by DNA detection techniques such as PCR [89]. Despite the sequencing of genetic loci (*RPB1*, *RPB2*, *D1/D2*) and the internal transcribed spacer (ITS) of ribosomal RNA (rRNA) being commonly used, especially in reference centres [58,74,90], different PCR endpoint trials, multiplex PCR [91], or PCR of Restriction Fragment Length Polymorphisms (RFLP) [89,92] could be more accessible in centres with economic or equipment limitations. Recently, two commercially available PCR assays, AurisID (OLM, Newcastle Upon Tyne, UK) and Fungiplex Candida Auris RUO Real-Time PCR (Bruker, Bremen, Germany) have been shown to reliably identify *C. auris*, even at low DNA concentrations [93].

In addition, many microbiology laboratories presumptively identify *C. auris* using chromogenic media, due to better accessibility and lower cost. Consequently, some media which allow for rapid screening after 24 h of incubation have been created, such as HiCrome *C. auris* [94]. Furthermore, the culture medium CHROMagar *Candida*, complemented with Pal agar [56], has been shown to be useful in the differentiation of *C. auris* from *C. haemulonii*. Due to its triazole resistance, the use of high concentration fluconazole as media supplementation could optimise the presumptive recognition of *C. auris* in higher prevalence zones which lack easy access to definitive identification techniques [95].

### 4.4. Virulence

Since *C. auris* became a major public health problem, efforts have been devoted to investigating the pathogenicity degree of several clones, strains, and worldwide isolates of *C. auris.* Nevertheless, data on its virulence compared to other *Candida* species, as well as on its phenotypical, morphological, or molecular pathogenicity determinants, are still limited.

*C. albicans* is considered the most virulent species of the *Candida* genus [70,71,96]. *Candida* species express several pathogenicity factors that contribute to their pathogenicity and virulence within the host. Among them, it is important to highlight the synthesis of molecules such as phospholipases, aspartic-proteases, or molecules related to the recognition of host proteins that increase tissue adhesins, and morphogenesis, as well as a phenotypic switch to a filamentous phenotype, enabling higher adaptability to intrahost changes [96]. 

Despite *C. auris* initially being considered unable to filament in vivo or, in any case, only able to produce rudimentary pseudohyphae under stress [75,76], some works using strains from different origins and clones have described an in vivo virulence similar or even greater than that of *C. albicans* [43,62,97]. Nonetheless, the results of the few studies on the pathogenicity of *C. auris* are relatively diverse, as seen in Table 1 [43,61,62,63,64,65,97]. Differences have been noted, not only in comparison with other species of the genus, but also regarding different clones, strains, and individual isolates. Further studies are, hence, needed, using a larger number of strains from different geographical regions, clinical isolates, and clades [50,61,65,98].

During the last several years, several research groups have analysed the pathogenicity differences of *C. auris* in comparison to other *Candida* species. Different models have been used: from in vitro studies assessing different transcriptional profiles from strains with different phenotypes [101], to animal models with a diverse complexity. These include invertebrate models in *Caenorrhabditis elegans* [67,99], *Drosophila melanogaster* [100], and the recently popularised model in wax moth larvae, *Galleria mellonella* [43,61,62,63,64,65,66,67], as well as vertebrates such as the traditional murine model [64], and, more recently, the zebrafish *Danio rerio* [97].

*G. mellonela* has recently gained importance in the study of fungal pathogenesis and, especially, *Candida* spp. virulence. Owing to the functional and structural similarity of the larval innate immune system to that of mammals, its low cost, as well as the possibility of working with larger samples in short timeframes thanks to its short vital cycle and, importantly, due to the lack of ethical implications involved, its popularity has been increasing recently [61,71,102,103,104,105,106,107]. 

The first data of experimental pathogenicity of *C. auris* came from the studies of Borman et al. [43], using 12 isolates from the United Kingdom outbreak. They showed more aggregative phenotypes of *C. auris* to be in vivo than non-aggregative strains. Moreover, the first were considered almost as virulent as *C. albicans*, despite their striking inability to filament. In addition, Sherry et al. [62], who also used four different strains from the United Kingdom, documented that non-aggregative phenotypes of *C. auris* showed a higher lethality than *C. albicans* reference strain SC5314, using a standardised inoculum of 10^5^ colony forming units (CFU), while C. glabrata and aggregative *C. auris* were significatively less virulent. In a model of *C. elegans* using 37 *C. auris* strains from Venezuela [9], they also appeared to show a similar pathogenicity degree to *C. albicans*, but less virulence than *C. haemulonii* [99]. However, these results could not be reproduced using strains of other geographical origins.

The works of Carvajal et al. [66] and Muñoz et al. [64] analysed the differential pathogenicity using Colombian strains. The study of the first group in *G. mellonella* did not show significative differences in the virulence of aggregative and non-aggregative strains, with more than 50% of the strains being less lethal than the reference strain of *C. albicans* SC5314; these findings are similar to the results obtained by Romera et al. [63] with Spanish isolates, also in *G. mellonella*. The second group developed both a *G. mellonella* and a neutropenic murine model, and used *C. albicans* SC5314 and ATCC10231 strains as a high pathogenicity control, and *C. haemulonii* as a low virulence control. Despite *C. auris* phenotypes not being determined, the four strains used showed a significant intermediate lethality between *C. albicans* and *C. haemoulonii* in *G. mellonella*, as reported by Garcia-Bustos et al. [61], but these results were not replicated in the murine model.

Therefore, this heterogenicity in intra- and interspecific virulence advocates for the hypothesis that the morphogenetic variability is an inherent trait of *C. auris*, and an indicator of its flexibility and adaptability to different environments and stimuli [61], particularly after some authors induced aggregation after exposition to triazoles and echinocandins [108].

This potential ability to phenotypically switch may result from a survival mechanism outside of the host. In fact, isolates from environmental and epidemiological surveillance samples more frequently presented an aggregative phenotype. Moreover, they demonstrated a greater ability to form biofilm structures; both traits related to the difficulty for their definitive eradication in the health environment and in colonised patients [109,110]. In addition, replicative aging resulting from asymmetric cell division has been shown to cause further phenotypic differences, and older *C. auris* cells have been associated with increased virulence in *G. mellonella* [111]. 

The pathogenicity determinants of *C. auris* are not completely clarified. The formation of biofilms and filamentation constitute two of the main virulence factors of *Candida* species. Other important factors have been described, such as phenotypic switch, metabolic flexibility and adaptation to different pH, production of extracellular hydrolytic and cytolytic toxins, heat shock proteins (HSP), and development of adherence and recognition mechanisms of surfaces and host cells [112,113]. 

As previously stated, *C. auris* is able to filament both in vivo and in vitro [61,75,76,77]. However, the pathogenic implication of hyphae or pseudohyphae formation in *C. auris* is still unknown. Some studies have not been able to demonstrate the expression of proteins related to the formation of these structures, such as the candidalysin (ECE1) or hyphal cell wall protein (HWP1) in certain *C. auris* strains [49]. Yue et al. [75] analysed the expression profile of genes related to the regulation of filamentation, and discovered similarities with *C. albicans,* showing an increased expression of genes implicated in hyphae formation such as *HGC1*, *ALS4*, *COH1*, *FLO8*, *PGA31*, and *PGA45* in filamentous strains, with regard to strains that only showed yeast-form structures.

*C. auris* is able to form biofilms, a trait which also constitutes a major challenge in clinical practice. The colonization of surfaces in patients undergoing any type of instrumentalisation increases, on the one hand, the risk of invasive candidiasis and generating new outbreaks, and decreases, on the other hand, the possibility of eradicating patient colonisation. A large number of IFI cases due to *C. auris* have been described related to health devices, such as urinary tract infections (UTI) in patients with indwelling catheters, cardiovascular infections, or neurosurgical instrument-related infections [16,17,18,114,115]. The *C. auris* tendency to form biofilms in human skin as well as in animal skin models with an elevated microbiological burden [116] has been related to an increased expression of adhesins (*IFF4*, *CSA1*, *PGA26*, *PGA52*, *PGA7*, *HYR3*, and *ALS5*) [117], with differential regulation based on the biofilm maturity [117,118]. In addition, biofilms also influence drug resistance by physical means, by hindering drug penetration in the most isolated regions of the dense biofilms [117,119], and expressing genes related to biofilm with added efflux pump action or glucan modifier enzyme action [117,119,120].

Some genomic studies have demonstrated that *C. auris* shares some of the pathogenicity determinants with other species of *Candida*, such as secretion of aspartic-proteases (SAP), lipases, phospholipases, and YPS proteases [67,69]. Other virulence factors include the expression of oxidoreductases, transferases, hydrolases [67], and haemolysins [121].

Finally, immune evasion has recently been considered an important trait of *C. auris.* Beyond phenotypic plasticity, some works have reported the ability of this fungus to evade neutrophil attack and effective phagocytosis both in human and animal models [116,122]. This finding is in line with previous clinical works, suggesting that neutropenia is not an important risk factor for invasive candidiasis by *C. auris* [61].

### 4.5. Antifungal Resistance

An important proportion of *C. auris* strains are resistant to multiple, and in some cases, all available antifungal treatments used in clinical practice. The estimated frequency of resistance to fluconazole, amphotericin B, and echinocandins surpasses 90%, 30%, and around 5%, respectively, according to CDC tentative breakpoints [10,123]. As a consequence, the management of IFI caused by *C. auris* is extremely complicated.

As with other microbiological characteristics of the species, the degree of drug resistance is highly variable between strains [10,123,124]. Different isolates also show variable susceptibility profiles to triazoles, even within the same clade [10,125,126], evidencing high regional, intra-, and inter-clade diversity.

Ergosterol is the main component of the fungal cell wall, and is synthesised by lanosterol demethylase enzyme, coded by the *ERG11* gene. Ergosterol is the target of polyenes such as amphotericin B, and the enzyme 14-alpha demethylase is the target azole drugs [10,127]. Several mutations have been detected in this gene, which partially explains the resistance to triazole drugs of *C. auris* (F126L, Y132F, VF125AL, and K143R) [19,126]. These mutations frequently coexist with others in resistance-related genes, such as *MDR1*, *CDR1*, *YMC1*, and *TAC1B* [128,129,130]. Less frequently, mutations in the *FKS1* gene (S639F and F635Y) and in the chitin synthase gene *CHS1* have been documented [131]. *FKS1* codes the catalytic subunit of the synthase of 1,3-beta-D-glucane—the target of echinocandins—which bestows its resistance [124]. Moreover, mutations in the gene *CHS1* were also found to cause echinocandin resistance [131].

Flucytosine is a nucleoside analogue that inhibits fungal nucleic acid synthesis, and has been frequently used in combination regimes to treat severe invasive infections by *C. auris*, especially those associated with external devices. The pro-drug is activated by fungal uracil-phosphoribosyltransferase, encoded by the *FUR1* gene [132]. Unlike with other *Candida* species, an F311I substitution in the *FUR1* gene has been reported in a flucytosine-resistant *C. auris* strain [123].

Furthermore, beyond molecular mechanisms of antifungal resistance in *C. auris*, phenotypic modifications and biofilm formation also influence the degree of drug resistance [61,108,110]. As previously stated, replicative aging resulting in asymmetric cell division and phenotypically distinct daughter cells has been associated with increased virulence, but also drug resistance [111,133]. Senescent *C. auris* cells showed a higher tolerance to fluconazole, micafungin, flucytosine, and amphotericin B. Bhattacharya et al. [111] demonstrated that fluconazole resistance in these cells was due to gene duplication in *CDR1* and *ERG11.* Both high- and low-density *C. auris* biofilms are associated with high levels of resistance to azoles, amphotericin B, and micafungin [13]. Biofilms confer pharmacodynamic resistance to antifungal agents related to low penetration in infected tissue after parenteral administration, but they can also overexpress ATP-binding cassette (ABC) and major facilitator superfamily (MFS) transporters. The resistance to triazoles has been shown to be increased by 4-16-fold in these cases [117]. The presence of glucan and mannan polysaccharides able to sequestrate antifungal drugs, the protection of cells from oxidative stress by the extracellular matrix, and phenotypic plasticity culminate in a rise in the drug resistance in this yeast to voriconazole by 4-fold, amphotericin B by 20-fold, and to micafungin by 60-fold [13].

To date, the highest rates of multidrug resistance have been described for clade I isolates, in regions from United Kingdom, India, and Pakistan. Resistance to azoles in strains from clade I rises up to 97%, and more than 50% of isolates show resistance to amphotericin B, or present resistance to two or more antifungal agents [38]. Strains from Venezuela belonging to clade IV also exhibit amphotericin B resistance rates of approximately 50%, together with the highest rate of resistance to echinocandins, such as micafungin, of around 7% [38]. This rate is similar to that observed in Spanish isolates from clade III [17,134]. Contrarily, strains from clade II show fluconazole resistance from 11–14% [13,119].

### 4.6. Persistence in the Healthcare Environment

*C. auris* shedding between patients is widely facilitated by its capability of persisting in viable forms in the environment for long periods of time [135]. Viable yeasts have been recovered from surfaces in rooms and bathrooms of the healthcare environment, such as mattresses, pillows, bed sheets, tables, sinks, toilet, walls, door and tap handles, as well as sanitary material such as temperature probes, blood pressure cuffs, mechanical ventilators and tubes, oxygen masks, and personal devices such as mobile phones [18,20,135,136,137,138].

Furthermore, it is able to survive on non-porous abiotic surfaces, such as plastic or steel, for up to 28 days [15,59]. 

## 5. Clinical Manifestations 

### 5.1. Colonization 

*C. auris* possess a unique capacity to be transmitted from patient to patient through contact. Contrary to other *Candida* species, this species is not naturally present in the human organism as saprophytic flora, and is not a common commensal in the gastrointestinal tract as can happen with other species such as *C. albicans* [139,140,141].

Despite its greater thermotolerance [41], *C. auris* is able to colonise multiple anatomical surfaces, showing special predilection for the skin, and being frequently found not only in the axilla or groin, but also in oropharyngeal or anorectal exudates. In addition, it is commonly isolated in urine, nasal fossa, and external auditory conduct, and it easily colonises wounds [17]. However, the tissue with the greatest permittivity to multiplication and transmission among hosts seems to be the skin [40]. After colonising the patient’s skin, *C. auris* yeasts produce a complex biofilm of multiple layers with high adherence and fungal burden, proliferating in regions which reproduce the humid conditions of sweat in folds and covered surfaces [116]. This happens to a greater extent than in other more commonly isolated species, such as *C. albicans.*

The presence of instrumentation and external devices in which *C. auris* can form biofilms increases the risk of multifocal colonisation, the persistence of this colonisation, and dramatically decreases the possibilities of its eradication.

The colonisation of patients in healthcare environments occurs rapidly, within hours after exposure, and can last for weeks, months, or even years [40,142], despite the use of agents such as chlorhexidine, nystatin, and echinocandins [20,143]. In fact, this persistent colonisation can lead to repeated IFI episodes over several months, regardless of an optimal antifungal therapy [142,144].

Additionally, Biswal et al. [20] reported hand colonisation in 3% of screened healthcare personnel. Therefore, proper handwashing protocols must be implemented in the clinical setting, as the colonisation of workers might be an important vehicle for *C. auris* transmission.

### 5.2. Infection

**Figure 2:**
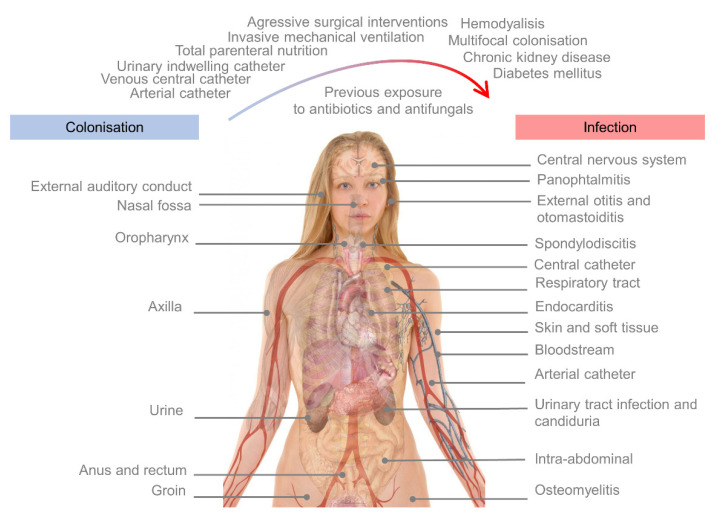
Schema representing the most common colonisation, invasive infection sites, and risk factors for deep-seated infections in patients colonised by *C. auris*.

Since its first description in 2009, *C. auris* has been isolated in multiple invasive locations. The clinical manifestation and the organic spectrum of IFI caused by *C. auris* do not particularly differ from other *Candida* species. As such, the candidaemia or bloodstream infection is the most common manifestation [135,145]. Many other infections associated with intravascular devices have also been described, such as central catheters, endocarditis [17,74,115,146], urinary tract infections or candiduria [142,144], central nervous system infections [147], respiratory tract infections [15,49,62,74], intra-abdominal infections [17,148], skin and soft tissue infections [55,58], external otitis [6,134], otomastoiditis [44], panophthalmitis [149], and even osteomyelitis and spondylodiscitis [83,150] (Figure 2). In fact, in our institution (University and Polytechnic Hospital La Fe, Valencia, Spain), *C. auris* colonisation was considered a temporary absolute contraindication for cardiac transplantation, due to high rates of cardiovascular infection and death.

### 5.3. Risk Factors of Invasive Fungal Infection in C. auris Colonised Patients 

Due to the high colonising power of *C. auris*, the scientific community has overturned studying the risk factors for the development of candidaemia or IFI, especially in colonised patients of critical care units. Early identification of risk factors may help to estimate the risk of candidaemia, and identify a high-risk population that could benefit from early or prophylactic antifungal treatment, as well as control of modifiable risk factors.

The risk factors do not greatly differ from those of other *Candida* species, and include the presence of not only central venous catheters, arterial catheters, indwelling catheters, but also conditions such as chronic kidney disease, total parenteral nutrition (TPN), diabetes mellitus (DM), haemodialysis, invasive mechanical ventilation, aggressive surgical interventions, sepsis, multifocal colonisation, and previous exposure to antifungals or antibiotics [16,18,20,151,152,153]. In a previous study undertaken by our group, we developed and validated a predictive model of candidaemia in critically ill colonised patients through a prospective approach, creating an equation that could help to predict the estimated risk of candidaemia, dependent on the present risk factors [151]. TPN (adjusted OR 3.73), previous surgery (adjusted OR 1.03), sepsis (adjusted OR 1.75), previous exposure to antifungal agents (adjusted OR 1.17), arterial catheters (adjusted OR 1.46), central venous catheters (adjusted OR 1.21), presence of advanced chronic kidney disease (adjusted OR 1.35), and multifocal colonization (adjusted OR of unifocal colonisation 0.46) were proven to be independent predictors of candidaemia. 

The most common colonisation sites, invasive infection sites, and risk factors are graphically represented in Figure 2.

### 5.4. Complications, Mortality, and Prognosis

Candidaemia is the most frequent severe manifestation, and its complications depend on a constellation of several diverse and related factors. Some of these factors include pathogen traits, such as resistance pattern or strain virulence, factors dependent on the infection itself, such as its origin or its extension, and host factors, such as comorbidity and therapeutic limitations due to pharmacodynamic or pharmacokinetic parameters, and drug availability.

Patients with candidaemia can develop fungal blood dissemination to different organs, producing a generalised infection, septic metastasis, septic shock, multiorgan failure and, finally, death on many occasions, despite optimal antifungal therapy. Moreover, colonisation and localised infections can lead to the appearance, perpetuation, or relapse of candidaemia months after adequate treatment [16,18,20,40,142,144]. 

The mortality of invasive infections caused by *C. auris* is comparatively larger than the majority of *Candida* species, and varies from 30% to more than 70%, according to the available series [7,13,16,18,19,20,134,147]. Early detection and prompt treatment are associated with greater survival.

## 6. Treatment of Invasive *C. auris* Infections

The greater problem in the management of *C. auris*-infected patients is the multi-, and on some occasions, pandrug resistance of the isolates, which confer a high therapeutic failure rate with all types of antifungal treatments [154].

According to the CDC recommendations [155], and to the limited available data in absence of clinical guidelines, echinocandins are the initial recommended treatment for *C. auris* infections. Despite the fact that many of the isolates are susceptible to this therapeutic group, resistance is easily acquired, and the incidence of strains with decreased susceptibility is increasing rapidly [134]. Patient monitoring must be close, and if there is no clinical or microbiologic response within five days of the initial treatment, echinocandins should be changed for amphotericin B, or amphotericin B should be added in a combination regime [155]. The therapeutic measures must be accompanied by a judicious management of antimicrobials, medical devices, and instruments, as well as an adequate control of the infectious foci.

There is growing evidence of the efficacy and effectivity of combination regimes, using echinocandins with a second or even third drug, such as amphotericin B, flucytosine, voriconazole, or isavuconazole, among others [156,157]. Less frequently, combinations with other drugs such as co-trimoxazole [158], lopinavir [159], aprepitant [160], or suloctidil [161] have been used. However, many of these results have been obtained in vitro, and need to be further validated in vivo.

Nevertheless, multidrug resistance in *C. auris* has urged research for new molecules with antifungal activity, as society will face a foreseeable scenario of more complex infections with a higher rate of therapeutic failure. Some of these new compounds are detailed in Table 2.

## 7. Prevention and Control of *C. auris*

All of the factors previously described show that *C. auris* combines all of the essential characteristics for a pathogen to pose a threat to public health:

- Potential to spread through horizontal transmission and to cause outbreaks;
- Ability to cause serious and life-threatening infections;
- Multi-resistance profile and limitations for optimal treatment.

Currently, and as defined by the CDC in its report on antibiotic resistance threats in the United States of 2019, *C. auris* is considered a public health threat that requires urgent and aggressive action, together with carbapenem-resistant *Acinetobacter*, and carbapenem-resistant Enterobacteriaceae, among others. Therefore, the measures for control and prevention are similar, and based on previous protocols.

All potential sources and reservoirs should be detected, and *Candida* identification should be performed at a species level, even from non-sterile sites [42,135]. Clinicians should also be directly alerted after *C. auris* isolation, and further retrospective case-finding studies should be ideally performed, in order to improve the traceability of cases.

The patient must be immediately isolated and identified, and all health assistance must be performed following transmission-based precautions and use of personal protective equipment, with special precautions when appropriate. Negative pressure rooms have been proposed when available for *C. auris* colonised patients [135]. We recommend the periodic reassessment of colonisation on a weekly basis, through the performance of epidemiological surveillance cultures from the axilla, groin, oropharyngeal exudate, and anal and rectal samples. Invasive samples should be taken in an individualised manner, especially in patients undergoing surgical interventions, or any kind of instrumentation.

Patients, and healthcare personnel in close contact with them, should be screened for *C. auris*, and appropriate hand hygiene programs should be implemented for all healthcare personnel in potential contact with *C. auris*-colonised patients. 

Environmental cleaning should be performed regularly, preferably preferably sodium hypochlorite or hospital grade sporicidal disinfectants, at a minimum of 4–5 times a week. Single-patient devices and patient-use items should be utilised when feasible, and all shared medical equipment should be thoroughly disinfected according to the manufacturer instructions [135]. In an outbreak setting, environmental cultures should be performed weekly.

In patients with known risk factors for candidaemia, skin and mucosal decontamination with chlorhexidine should be considered, and all manipulations must be performed following adequate care bundles and protocols.

Programs for the optimisation of antibiotic and antifungal stewardship are encouraged to be implemented in all facilities with *C. auris* isolations.

## 8. Conclusions

*C. auris* has unprecedently emerged as a multidrug resistant pathogen, with an alarming increase in the incidence of nosocomial outbreaks with staggering mortality and transmission rates, that has put health authorities and institutions worldwide in check for more than a decade now. Astonishingly, its emergence has been independent and simultaneous in several continents. Despite the fact that the scientific community has devoted efforts to exploring its biological traits, there is little evidence on its pathogenicity and the complex host–pathogen interactions. Due to its unique features not observed in other yeasts, it has been categorised as an urgent threat by the Centers for Disease Control and Prevention and other international agencies concerned with disease control. Furthermore, the current SARS-CoV-2 pandemic has created the ideal conditions for its spread, allowing it to become a lurking threat for COVID-19 patients, and several outbreaks of *C. auris* invasive infections have been reported in COVID-19 wards. Progress in its identification has been optimal, but much remains to be done to guarantee equity in lower-income countries, which often have limitations for the use of definite diagnostic molecular or spectrometry tools. Contrarily, the treatment of severe and complex invasive infections still takes a shot in the dark, and further strategies need to be assessed with novel drugs and combination regimes for enhancing outcomes. Improving control measures, early diagnosis, knowledge of risk factors for invasive infection, antifungal stewardship, and education of healthcare providers is needed to contain the threat of *C. auris*, a timely reminder that pathogenic fungi deserve equal attention in the new era of emerging infectious diseases.

## Figures and Tables

**Table 1:** Virulence of *C. auris* in different experimental animal models.

Organism	Virulence Results	Reference
*C. elegans*	*C. hameulonii < C. auris* = *C. albicans*	[99]
*C. elegans*	Non-Ag *C. auris >* Ag-*C. auris*	[67]
*D. rerio*	*C. auris > C. albicans > C. haemulonii*	[97]
*D. melanogaster*	*C. auris > C. albicans* Non-Ag *C. auris =* Ag *C. auris > C. albicans*	[100]
*G. mellonella*	*C. albicans > C. auris > C. parapsilosis* Non-Ag *C. auris >* Ag-*C. auris* Non-invasive isolates = invasive isolates	[61]
*G. mellonella*	*C. auris ≥ C. albicans* Non-Ag *C. auris >* Ag-*C. auris*	[43]
*G. mellonella*	Non-Ag *C. auris ≥ C. albicans* and *C. glabrata* Ag-*C. auris = C. glabrata*	[62]
*G. mellonella*	*C. auris < C. albicans* Non-ag *C. auris* = Ag *C. auris*	[66]
*G. mellonella*	*C. auris < C. albicans* Non-ag *C. auris* = Ag *C. auris*	[63]
*G. mellonella*	*C. albicans > C. auris > C. haemulonii*	[64]
*G. mellonella*	Non-Ag *C. auris >* Ag-*C. auris* Blood isolates *>* respiratory and urine isolates	[67]
Neutropenic *Mus musculus*	*C. auris* = *C. haemulonii*	[64]

Non-ag: non aggregative; Ag: aggregative.

**Table 2:** Novel drugs, molecules, or compounds with proven activity against *C. auris*.

Antifungal Agents	Reference
Fosmanogepix (APX001A)	[162]
Arylamidine (T-2307)	[163]
Ibrexafungerp (SCY-078)	[164]
Nitroxoline	[165]
PC945	[157]
VT-1598	[166]
**Other compounds**	
Carvacrol	[167]
Crotamine	[168]
Ebselen	[161]
Farnesol	[169]
Histatin 5	[170]
Rocaglates	[171]
